# Supplementary material for: Conductance stable and mechanically durable bi-layer EGaIn composite-coated stretchable fiber for 1D bioelectronics
Source: Nat Commun. 2023 Jul 13;14:4173. doi: 10.1038/s41467-023-39928-x (PMC10345103; doi:10.1038/s41467-023-39928-x)
Supplement: Supplementary file 1 — Supplementary Information [file 41467_2023_39928_MOESM1_ESM.pdf]

Conductance stable and mechanically durable bi-layer EGaIn composite-coated stretchable fiber for 1D bioelectronics

Gun-Hee Lee[1,2,3,†], Do Hoon Lee[1,2,†], Woojin Jeon[3,†], Jihwan Yoon[4], Kwangguk Ahn[4], Kum Seok Nam[3], Min Kim[1], Jun Kyu Kim[1], Yong Hoe Koo[5], Jinmyoung Joo[5], WooChul Jung[1], Jaehong Lee[6], Jaewook Nam[4], Seongjun Park[3,7,8,*], Jae-Woong Jeong[2,7,*], and Steve Park[1,7,8,*]

[1] Department of Materials Science and Engineering, Korea Advanced Institute of Science and Technology (KAIST), 291 Daehak-ro, Yuseong-gu, Daejeon 34141, Republic of Korea

[2] School of Electrical Engineering, Korea Advanced Institute of Science and Technology (KAIST), 291 Daehak-ro, Yuseong-gu, Daejeon 34141, Republic of Korea

[3] Department of Bio and Brain Engineering, Korea Advanced Institute of Science and Technology (KAIST), 291 Daehak-ro, Yuseong-gu, Daejeon 34141, Republic of Korea

[4] School of Chemical and Biological Engineering, Institute of Chemical Processes, Seoul National University, 599 Gwanak-ro, Gwanak-gu, Seoul 08826, Republic of Korea

[5]Department of Biomedical Engineering, Ulsan National Institute of Science and Technology (UNIST), 50, UNIST-gil, Ulju-gun, Ulsan 44919, Republic of Korea

[6] Department of Robotics and Mechatronics Engineering, Daegu Gyeongbuk Institute of Science and Technology (DGIST), 333 Techno Jungang-daero, Daegu, 42988, Republic of Korea

[7] KAIST Institute for Health Science and Technology, 291 Daehak-ro, Yuseong-gu, Daejeon 34141, Republic of Korea

[8] KAIST Institute for NanoCentury, 291 Daehak-ro, Yuseong-gu, Daejeon 34141, Republic of Korea

†These authors contributed equally to this work.
*Corresponding authors: Seongjun P. (spark19@kaist.ac.kr), J.-W.J (jjeong1@kaist.ac.kr), Steve P. (stevepark@kaist.ac.kr)

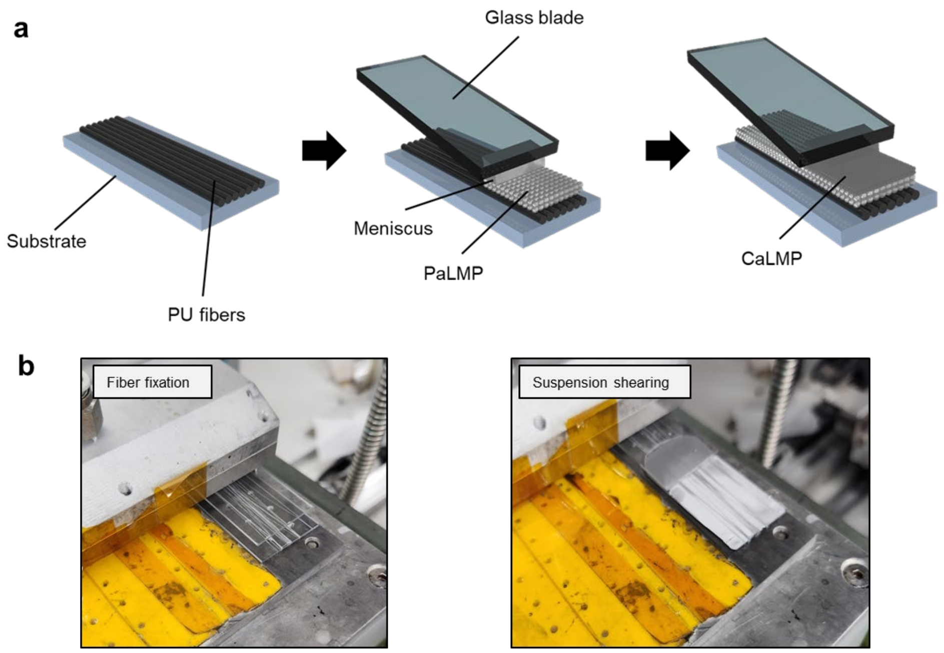

Supplementary Fig. 1| BiLMP coating on fiber. a, Schematic illustration of the BiLMP coating process on the fibers. **b**, Photograph of suspension shearing on fibers.

To fabricate the BiLMP-coated fiber, polyurethane fibers are fixed to the substrate. PaLMP film is coated with suspension shearing on the prepared substrate. Thereafter, CaLMP film is coated on the PaLMP film in the same way to make BiLMP-coated fiber.

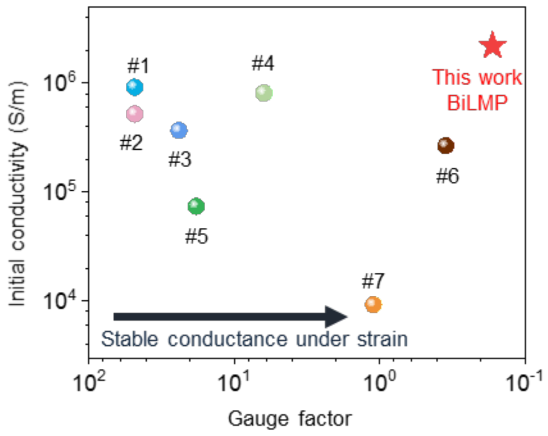

Materials	Initial conductivity (S/m) @ Ɛ = 0%	Maximum Strain	Gauge Factor	Ref (#)
**BiLMP, polyurethane fibers**	**2.2×10^6**	**150%**	**0.1**	**This work**
Ag NWs, polyurethane NFs	9.19×10^5	310%	*48.39	1
AgNPs, SBS fiber	5.22×10^5	140%	48.24	2
Ag flakes, fluoroelastomer, PVDF nanofibers	3.67×10^5	450%	*23.99	3
AgNWs, PDMS	8.13×10^5	80%	*6.25	4
Ag flakes, elastomeric fluorine copolymer	7.38×10^4	215%	*18.25	5
CNTs, PAAm	2.66×10^5	100%	0.35	6
AuNWs, SEBS	9.2×10^3	360%	*1.1	7

Supplementary Fig. 2| A comparison of this work to previous reported stretchable fiber electrodes.

Our deformable conductive filler (BiLMP) exhibits both high initial conductivity and the lowest gauge factor compared to previous referenced research studies..

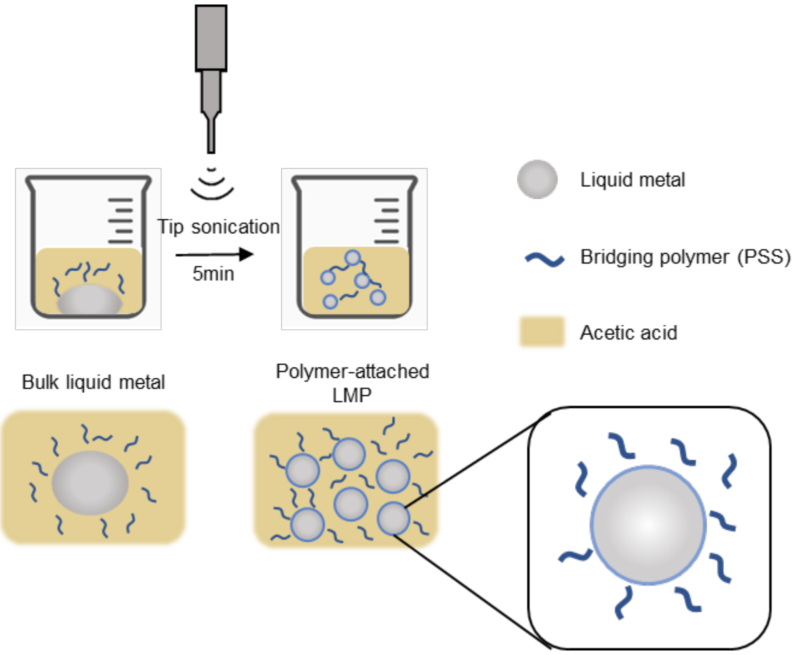

Supplementary Fig. 3| Schematic illustration of fabrication process of PaLMP ink.

To fabricate the PaLMP ink, PSS and bulk liquid metal are inserted in diluted acetic acid (AA) aqueous solvent (10 vol.%). To make uniform liquid metal micro particles, an acoustic field was applied for 5 min using a tip sonicator. During this process, PSS are attached to the liquid metal particles and are stabilized in the solution. The observed variance in Zeta potential values further supports this phenomenon. Specifically, the Zeta potential value of the LMP in diluted AA is measured at +53.2 mV, indicating a positively charged surface, while the PaLMP exhibits a Zeta potential value of -21.1 mV, suggesting a negatively charged surface. These distinct Zeta potential values serve as confirmation of the electrostatic interaction between the LMP and the negatively charged polymer.

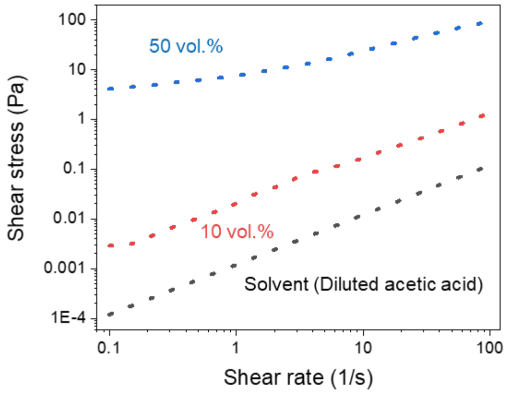

Supplementary Fig. 4| Viscosity curve of ink according to PaLMP concentration.

The addition of insoluble contents to the ink results in an increase in viscosity. At a concentration of 10 vol.%, the ink exhibits Newtonian behavior similar to that of bare solvent. However, at a concentration of 50 vol.%, the ink demonstrates non-Newtonian behavior with significantly increased viscosity. This high concentration of insoluble content leads to the formation of clusters, which is undesirable for solution shearing processes.

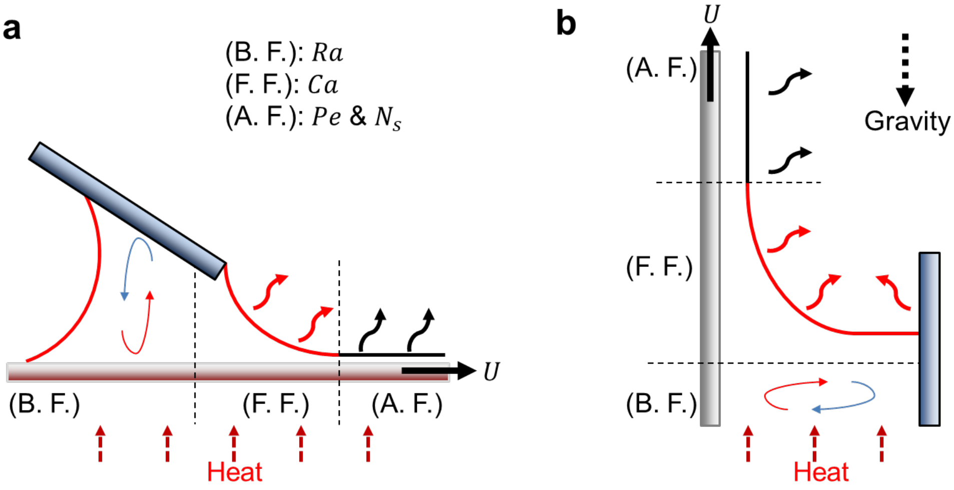

Supplementary Fig. 5| Scaling and rheological analyses. a, Schematic illustration of solution shearing. **b,** Schematic illustration of dip coating.

In this study, we undertook an evaluation and comparison of two distinct LMP coating techniques: solution shearing and dip coating, in a high evaporation rate coating system. Conventionally, a typical coating system comprises two stages, namely film formation and subsequent drying. However, in our LMP coating process, these two stages occur simultaneously due to the high evaporation rate. The coating velocities varied from relatively low[8] to high[9] values of 0.1-0.5 mm/s. The LMPs had an average size and volume fraction of 2 μm and 0.09, respectively. Notably, our findings reveal that solution shearing results in superior coating quality, while dip coating leads to poor coating quality and subsequent coating failure.

With respect to the coating failure observed with the dip coating method, we focused our attention on the primary differences between the two coating methods, namely heating and coating direction. In solution shearing, the substrate is heated, whereas in dip coating, the coating liquid pool is heated. Additionally, solution shearing is implemented in a direction perpendicular to gravity, while dip coating is performed parallel and opposite to gravity. Therefore, we conducted a further investigation of the interrelationship between these main differences and coating quality by examining the dimensionless numbers of the coating system for each coating method.

Supplementary Fig. 5 depicts significant dimensionless numbers for film formation (coating) and subsequent drying steps. Regarding the drying step after film formation, the Peclet (Pe) and Sedimentation (N_s) numbers may be considered[10,11,12]. However, in solution shearing, due to the high evaporation rate of the solvent in the LMP coating system, the particle distribution before film formation significantly affects the final coated film. Conversely, in dip coating, the particles are not entrained into the final coated film during film formation. Therefore, controlling film formation and particle distribution before film formation is essential to achieve a high–quality coating.

In this regard, we considered the Rayleigh (Ra) and capillary (Ca) numbers, which play a crucial role in determining the particle distribution before and during film formation, respectively. The numbers are defined as follows: $Ra = GrPr$, $Gr = g\beta(T_s - T_\infty)L^3/\nu^2$, $Pr = \mu C_p/k$, and $Ca = \mu U/\sigma$. Here, Gr and Pr are Grashof and Prandtl numbers, respectively. g is the gravitational acceleration. β and ν are the volumetric thermal expansion coefficient and kinematic viscosity of the fluid, respectively. L is the characteristic length scale of convective heat transfer. C_p and k are the specific heat capacity and thermal conductivity of the fluid, respectively. μ and σ are the viscosity and surface tension of the solvent, respectively. It is noteworthy that Ra is a type of Pe number and is widely used for systems in which heat transfer is considered[13,14].

With regard to Ra number, both coating methods have Ra numbers greater than an order of magnitude

of 10^3, which is a critical value for the onset of convection[15]. However, the Ra numbers did not exceed the order of magnitude of 10^9, a critical value over which unsteady turbulent flow may be developed[16]. Consequently, a turbulent convective mixing flow of particles was not observed in either coating method. Therefore, convective flow for particle distribution before film formation is not a critical factor in the coating–failure of dip coating.

Lastly, the film formation was discussed as the key factor for coating–failure of dip coating, and it was determined that the capillary number played a significant role[8]. Due to the small range of capillary numbers in dip coating, particles are not entrained upward by the moving substrate. According to Berteloot et al. [8], our capillary number range corresponds to the regime in which the formation of film thickness follows the Landau–Leivch model. Hence, for coating speeds of 0.1 and 0.5 mm/s, the resulting film thicknesses were calculated as 0.4 and 1 μm, respectively. Palma and Lhuissier[14] reported that particles do not become trapped in the coated film when their size exceeds the thickness of the film. It is noteworthy that the average particle size of 2 μm was considerably larger than the estimated film thickness, indicating that dip coating is not a suitable method for applying a large-sized LMP coating at given coating speeds. Furthermore, Supplementary Fig 4 demonstrates that the incorporation of particles does not significantly elevate the viscosity of the solution, making it impractical to effectively increase the capillary number in this system.

To sum up, our experimental results indicate that heating the coating system had no significant effect on both methods. Therefore, the heating and convective flow for the distribution of particles were not key factors for distinguishing coating quality of each method. Instead, film formation of the coated film was identified as the key factor, and previous analysis using the capillary number showed that particles are included and excluded in the solution shearing and dip coating, respectively. In conclusion, the solution shearing coating method was found to be more effective than dip coating for coating systems with a low range of capillary numbers, such as LMP coating with low coating speed and high evaporation rate.

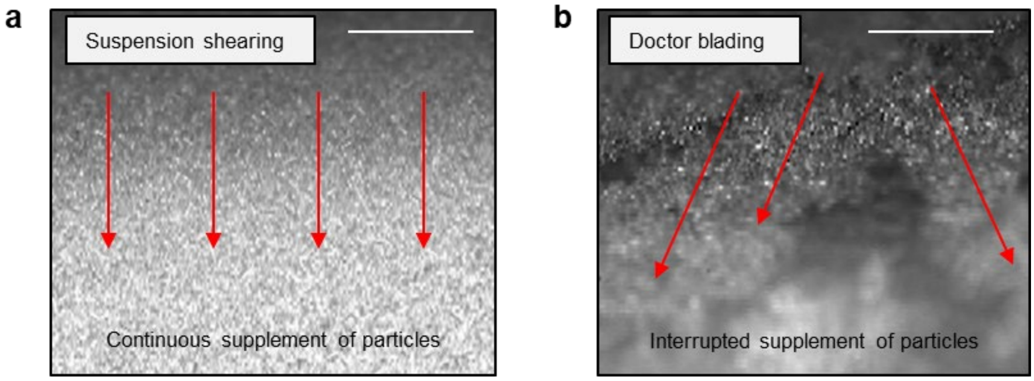

Supplementary Fig. 6| In situ optical microscope image during solution coating process. a, PaLMP ink coated through suspension shearing. Uni-directional continuous supplement of particles was observed **b**, PaLMP ink coated through doctor blading. Interrupted supplement of particles was observed. Scale bar: 50 μm

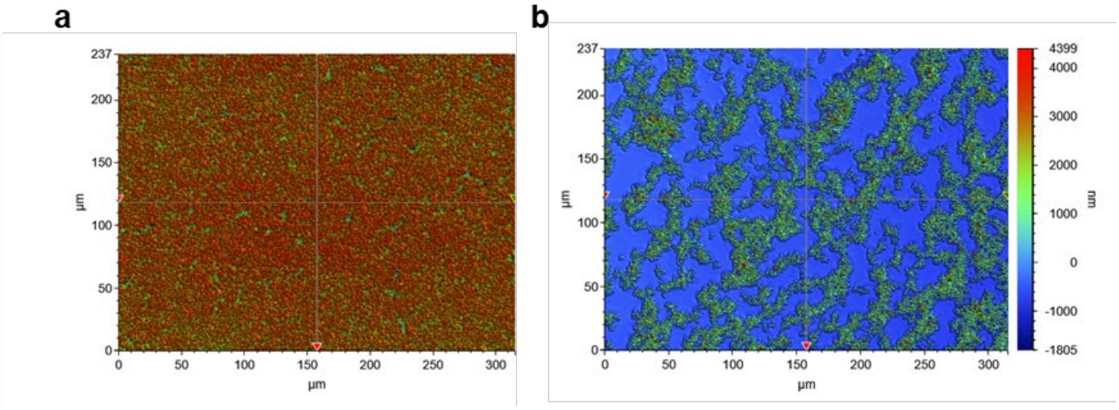

Supplementary Fig. 7| 3D surface profile images a, PaLMP film coated through suspension shearing. Compact and uniform assembly of particles was achieved **b**, PaLMP film coated through doctor blading. Coated film has several voids.

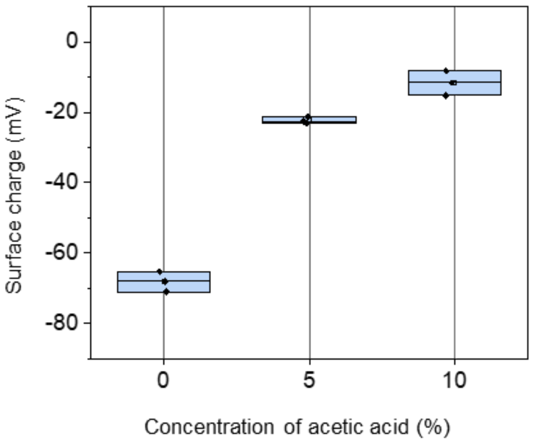

Supplementary Fig. 8| Zeta potential of PaLMP according to concentration of acetic acid. The values represent the mean, maximum, and minimum. (n = 3).

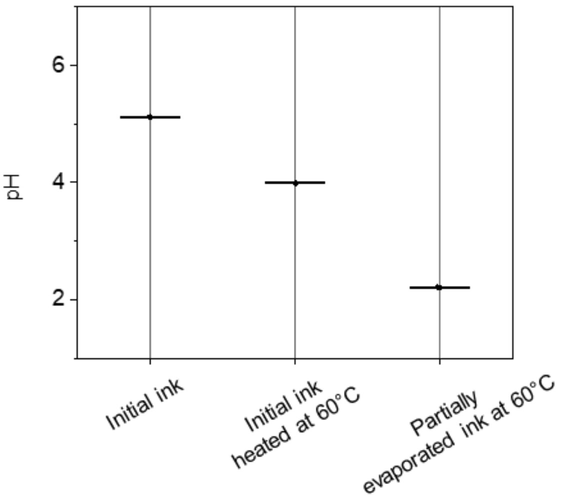

Supplementary Fig. 9| pH value of ink during heating and after evaporation.

As the solvent evaporates, the acidity of the ink increases.

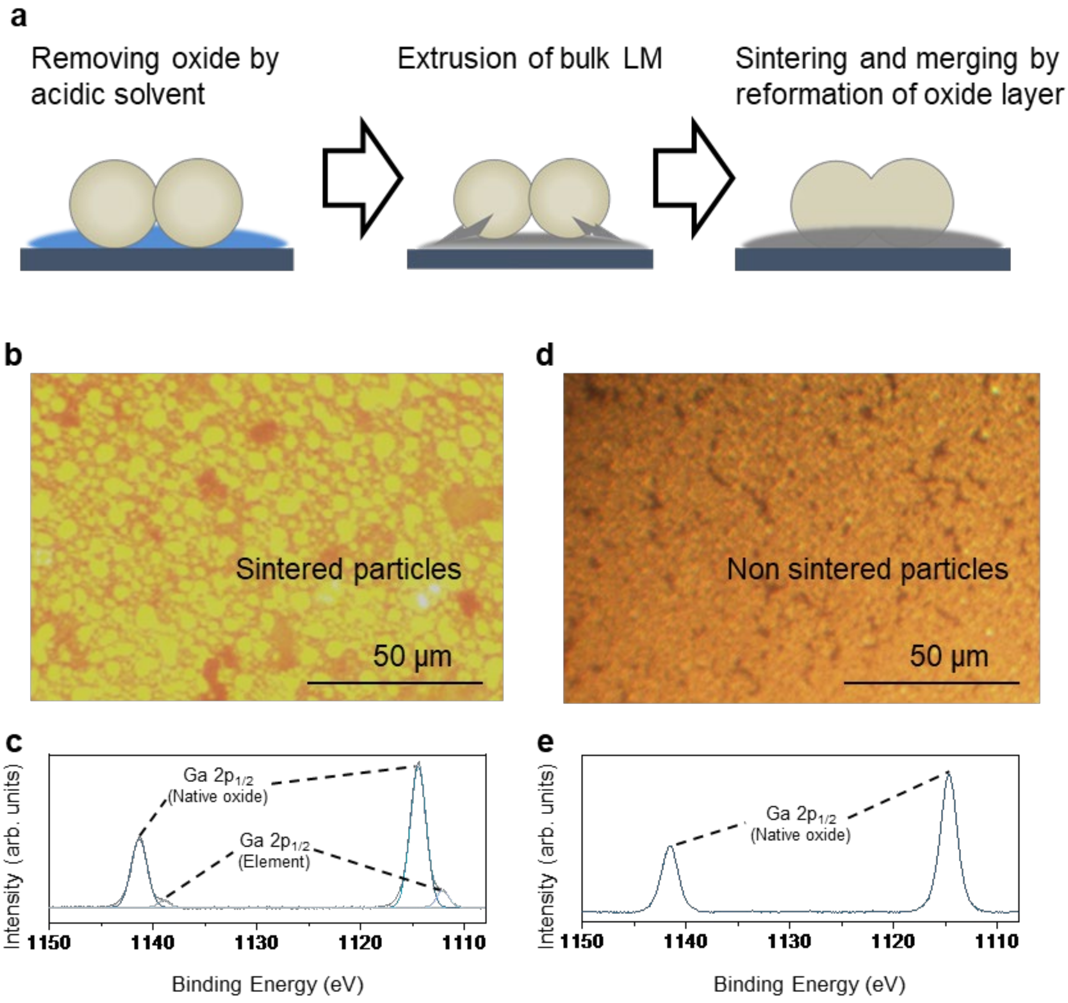

Supplementary Fig. 10| Chemical annealing of PaLMP. a, Schematic illustration of chemical annealing by acid during solution shearing. **b**, Bottom optical microscope image of coated PaLMP with acid. **c**, Gallium X-ray Photoelectron Spectroscopy (XPS) spectrum of PaLMP with acid. **d**, Bottom optical microscope image of coated PaLMP without acid. **e**, Gallium XPS spectrum of PaLMP without acid.

The process of suspension shearing involves evaporation of the solvent at an enlarged surface area in the meniscus, facilitated by heating. The ink used in this process is a diluted acetic acid (AA) mixed with water. As the boiling point of AA is higher than that of water, the acidity of the solvent increases during suspension shearing, which is evident from Supplementary Fig. 9. This increased acidity induces the chemical reduction of the surface oxide layer of the PaLMP. As the oxide is partially removed, the particles sinter, and the oxide layer naturally reforms at the surface. During this process, an oxide layer is formed at the interface between the LMP and the substrate, which acts as an adhesive layer, resulting in robust adhesion.

The bottom layer of the PaLMP film, as shown in the bottom OM image, exhibits a sintered particle morphology, indicating the successful particle sintering process. Additionally, the presence of a Gallium element peak in the XPS further supports our findings of consumption. On the other hand, in cases where there is no particle sintering, only an oxide peak is observed in the XPS spectra.

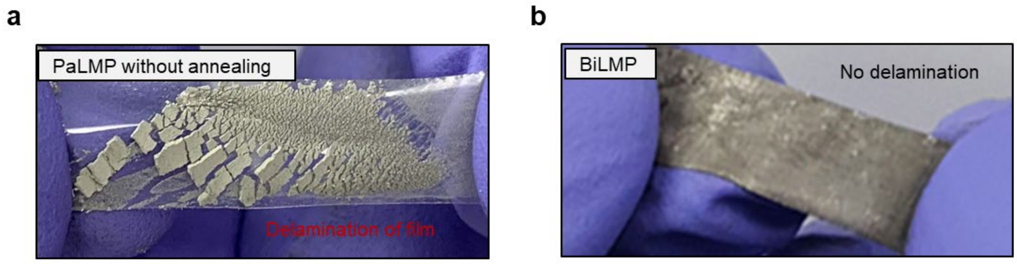

Supplementary Fig. 11| Adhesion between LMP and substrate a, Photograph of delamination of PaLMP film generated with solution without acetic acid. **b**, Photograph of BiLMP film under strain.

As discussed in Supplementary Fig. 10, the annealing process with an acidic solvent during the coating stage is crucial for achieving robust adhesion of the PaLMP film to the substrate. Without the presence of the acidic solvent, PaLMP coatings are prone to experiencing severe delamination when subjected to strain. In contrast, the incorporation of the acidic solvent during the annealing process of PaLMP results in robust adhesion, which effectively prevents delamination of BiLMP even under strain.

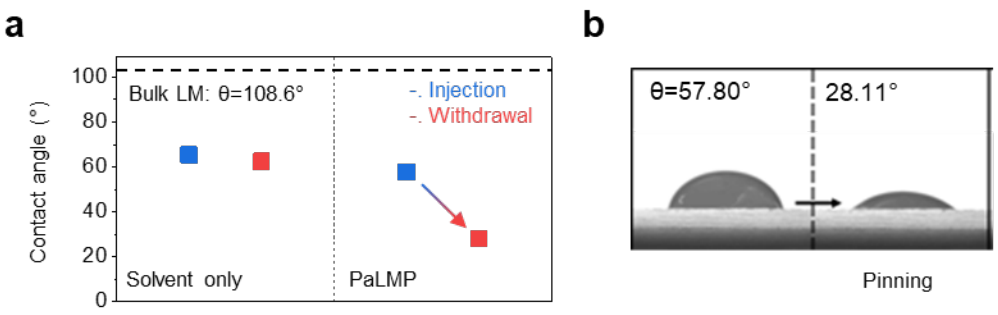

Supplementary Fig. 12| Wettability of ink a, Contact angle of PaLMP ink and its solvent (diluted acetic acid in water) on polyurethane substrate. Injection refers to the contact angle of the droplet and withdrawn refers to the contact angle of the droplet after partial removal of the droplet solution. The change in the contact angle upon solution withdraw means that droplet is pinned to the substrate surface. **b**, Photograph of PaLMP ink droplet before and after partial removal of the droplet solution.

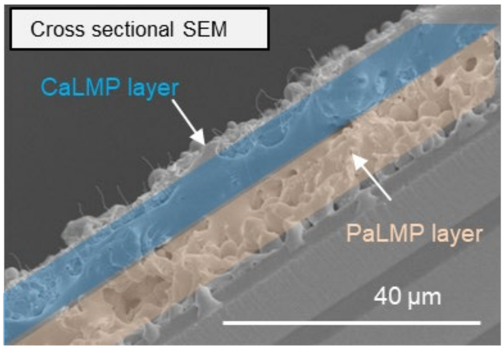

Supplementary Fig. 13| Cross sectional SEM image of BiLMP film.

The SEM image reveals the deposition of CaLMP onto PaLMP, resulting in the realization of a BiLMP film.

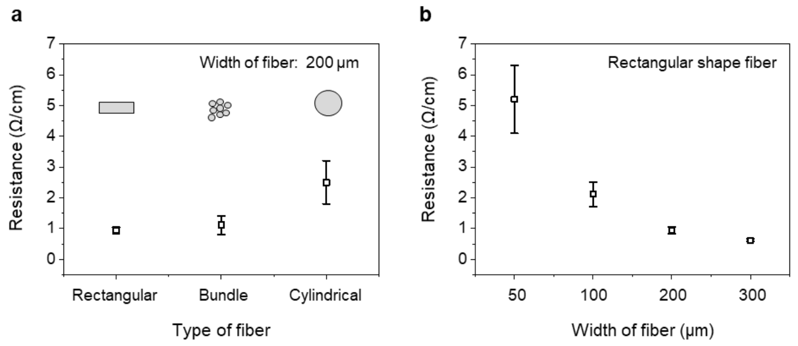

Supplementary Fig. 14| Resistance of BiLMP-coated fibers. a, Resistance of BiLMP coated fiber a ccording to type. **b**, Resistance of BiLMP coated recthangular shape fiber according to width. Values in a–b represent the mean and the 1.5 IQR (n = 6).

To achieve reliable electrical conductivity, it is recommended to use fibers with a width of over 50 μm.

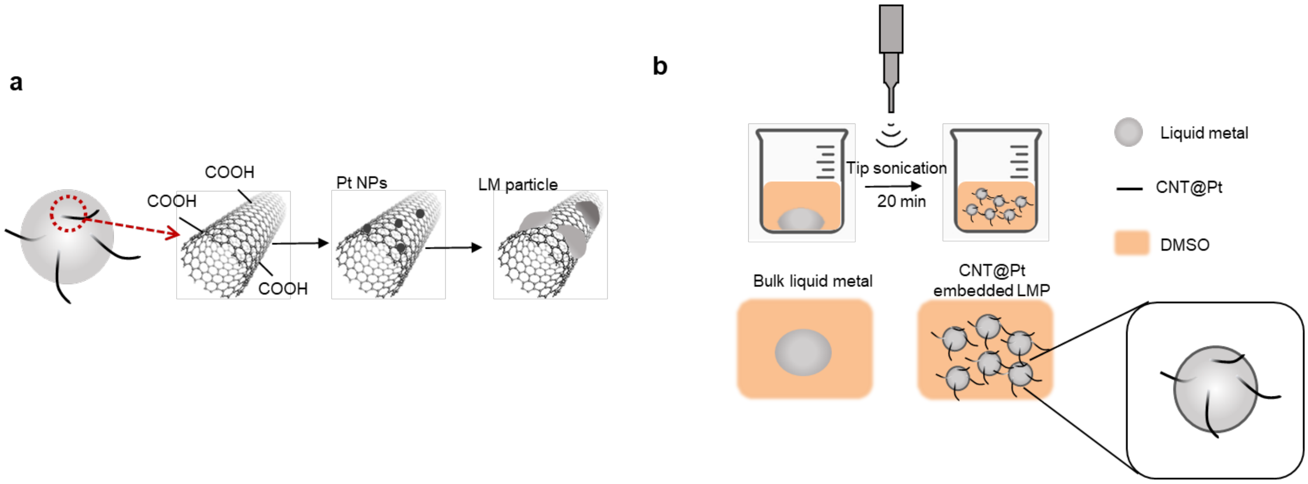

Supplementary Fig. 15| Schematic illustration of fabrication process of CaLMP ink. a, Fabrication process of Pt-decorated CNT (CNT@Pt) **b**, Fabrication process of CaLMP ink

Prior to the production of CaLMP ink, CNT@Pt is synthesized according to method in the previously reported work.[17,18] The synthesized CNT@Pt and bulk liquid metal are dispersed in dimethyl sulfoxide (DMSO) solvent. To make uniform liquid metal micro particles, an acoustic field was applied at an amplitude of 30% for 20 min using a tip sonicator. During this process, CNT@Pt are attached the liquid metal particle and stabilize the solution. The detailed fabrication process is in the method section.

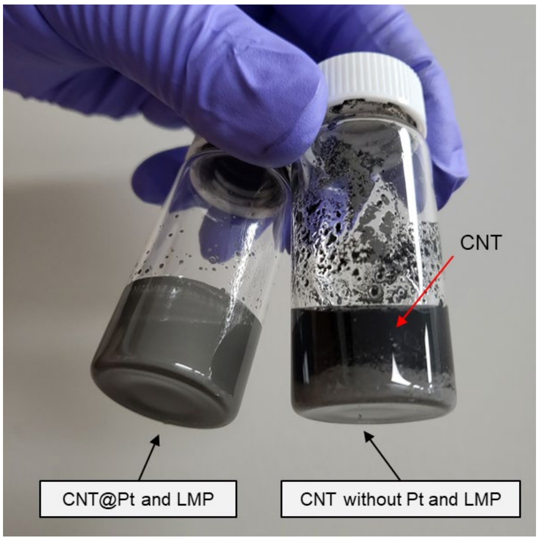

Supplementary Fig. 16| Photograph of CNT-LMP solution with and without functionalization of Pt.

The affinity of gallium-indium LM with metal facilitates its incorporation with Pt-functionalized CNTs. In the absence of Pt functionalization, CNTs and LM do not integrate well, leading to the formation of separate layers in the solution.

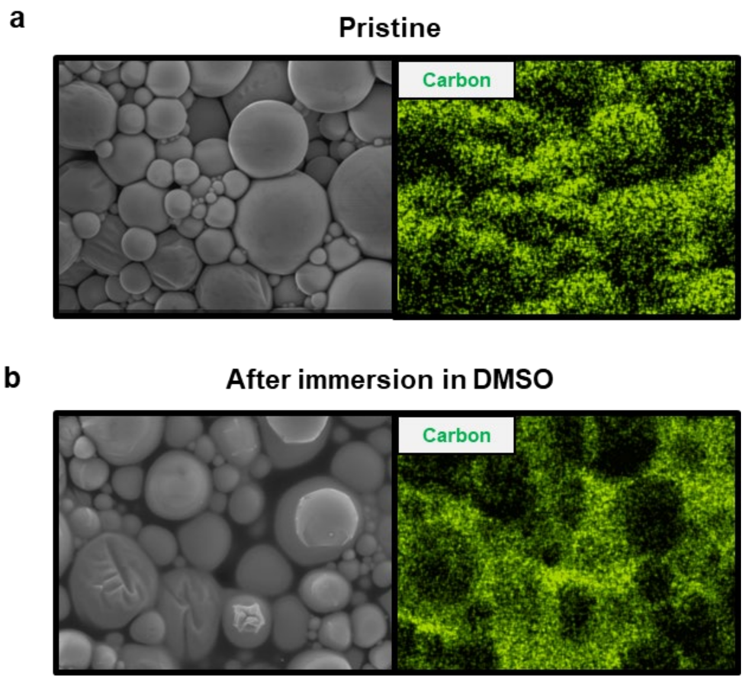

Supplementary Fig. 17| Energy-dispersive X-ray spectroscopy (EDS) of PaLMP. a, EDS mapping of carbon element in coated PaLMP. **b**, EDS mapping of carbon element in PaLMP after immersion in DMSO.

In the presence of a polar organic solvent, the electrostatic interaction between PSS and LMP is nullified, leading to the detachment of PSS from LMP. This detachment was confirmed by the observation of carbon element relocation, which is the main component of PSS, as indicated by EDS analysis.

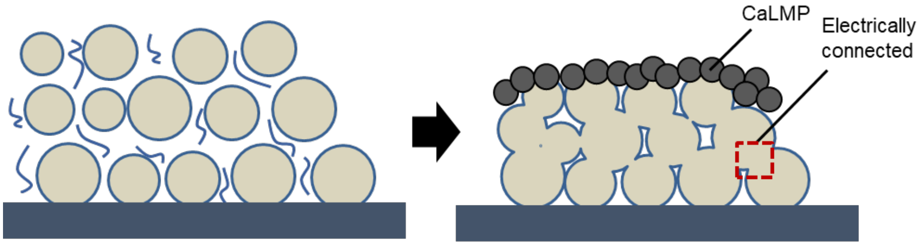

Supplementary Fig. 18| Schematic illustration of cohesion between PaLMPs induced by polar organic solvent.

The present study describes a facile approach for the attachment of a negatively charged polyelectrolyte (PSS) to the surface of LMP through electrostatic interaction, resulting in the formation of PaLMP. Immersion of PaLMP in a polar organic solvent such as DMSO (which is the solvent for the CaLMP ink) likely leads to the disruption of the electrostatic interaction and detachment of PSS from the surface. This process likely induces the rupturing of the oxide layer and further merging of the LMP, thus furthermore increasing the conductivity of the film, as described in Figure 3a in the main text. Moreover, the covering of merged PaLMP with CaLMP enhances the mechanical durability of the bi-layered film.

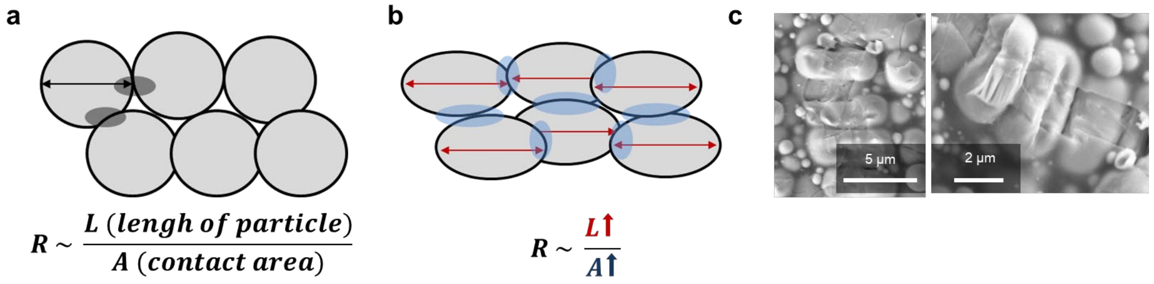

Supplementary Fig. 19| Elongation of deformable filler under strain. **a**, Schematic illustration of LMP without strain. Resistance is determined by the length of particles and contact area. **b**, Schematic illustration of LMP under strain. Increased length of particles compensates by an increase of contact area, which results in constant resistance value under strain. **c**, SEM image of LMP under strain. Elongation of particles was observed.

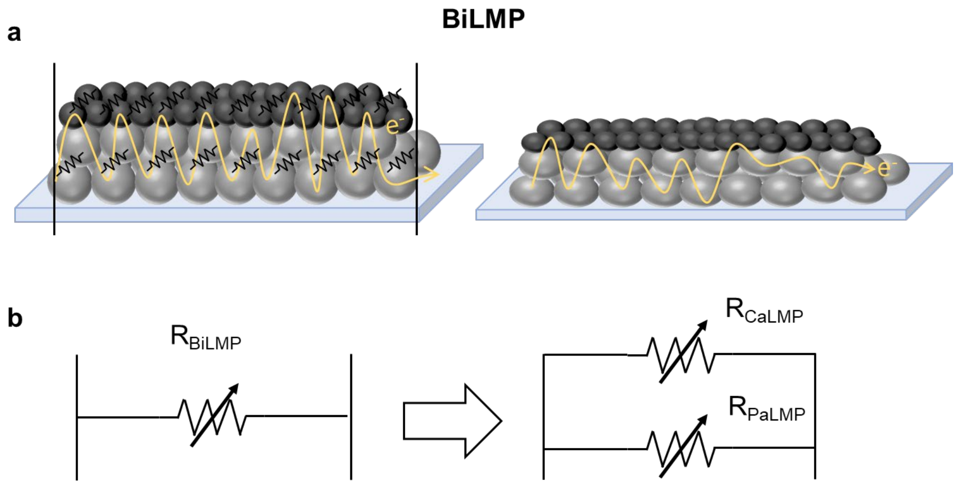

$$R_{BiLMP} = \frac{R_{CaLMP}R_{PaLMP}}{R_{CaLMP} + R_{PaLMP}} \; (R_{CaLMP} \gg R_{PaLMP}) \approx R_{PaLMP}$$

Supplementary Fig. 20| Equivalent circuit and mathematical modeling of BiLMP. **a**, Schematic illustration of BiLMP under strain. **b**, Equivalent circuit of BiLMP as parallel circuit of CaLMP and PaLMP.

The BiLMP structure can be effectively modeled as a parallel connection of CaLMP and PaLMP. Applying the principles of parallel resistors, we find that the overall resistance is primarily dictated by the resistor with the smallest resistance value. In our specific case, the sintered PaLMP layer demonstrates a lower resistance and exhibits excellent stability even under strain. This characteristic of the sintered PaLMP layer ensures that the overall resistance of the BiLMP structure does not experience a significant increase when subjected to strain. Thus, the BiLMP structure maintains its conductivity and reliability even under mechanical stress or deformation**.**

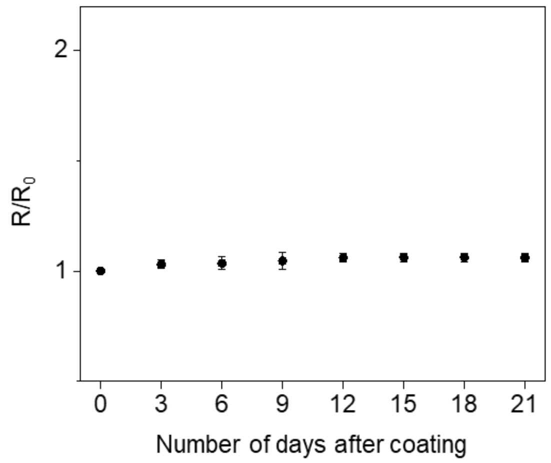

Supplementary Fig. 21| Resistance variation of BiLMP film with time.

The values represent the mean and the standard deviation ($n = 3$).

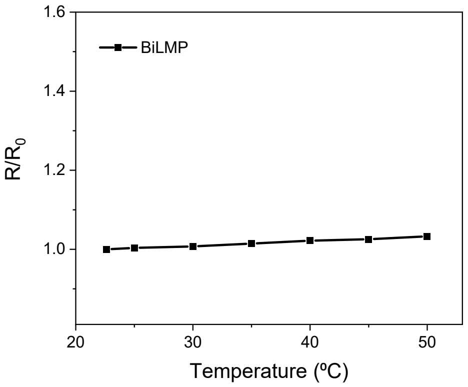

Supplementary Fig. 22| Resistance variation of BiLMP film according to temperature.

The resistance of BiLMP exhibits a slight increase with temperature, similar to conventional metals.

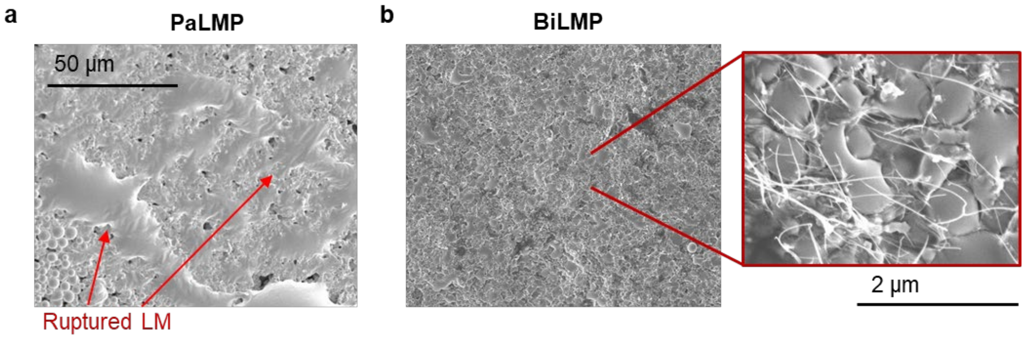

Supplementary Fig. 23| Mechanical durability of BiLMP film. **a,** Photograph and OM image of PaLMP film after application of shear force. **b**, Photograph and OM image of BiLMP film after application of shear force.

The top layer of PaLMP is covered with a thin polymer, which makes it prone to delamination upon the application of scotch tape. This delamination process results in severe rupturing and merging of the LM droplets within the film. However, when CNTs are incorporated into the BiLMP film and combined with chemical annealing, the mechanical durability of the film is improved. As a result, the BiLMP film does not experience delamination or rupturing of the LM droplets. The presence of CNTs and the chemical annealing process enhance the adhesion and structural integrity of the LM droplets within the film, leading to its improved mechanical durability compared to the PaLMP film.

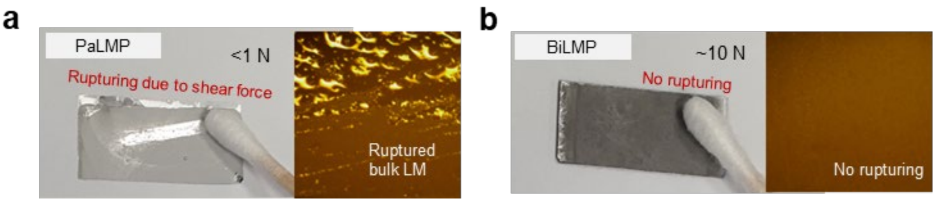

Supplementary Fig. 24| Mechanical durability of BiLMP film. **a,** Photograph and OM image of PaLMP film after application of shear force. **b**, Photograph and OM image of BiLMP film after application of shear force.

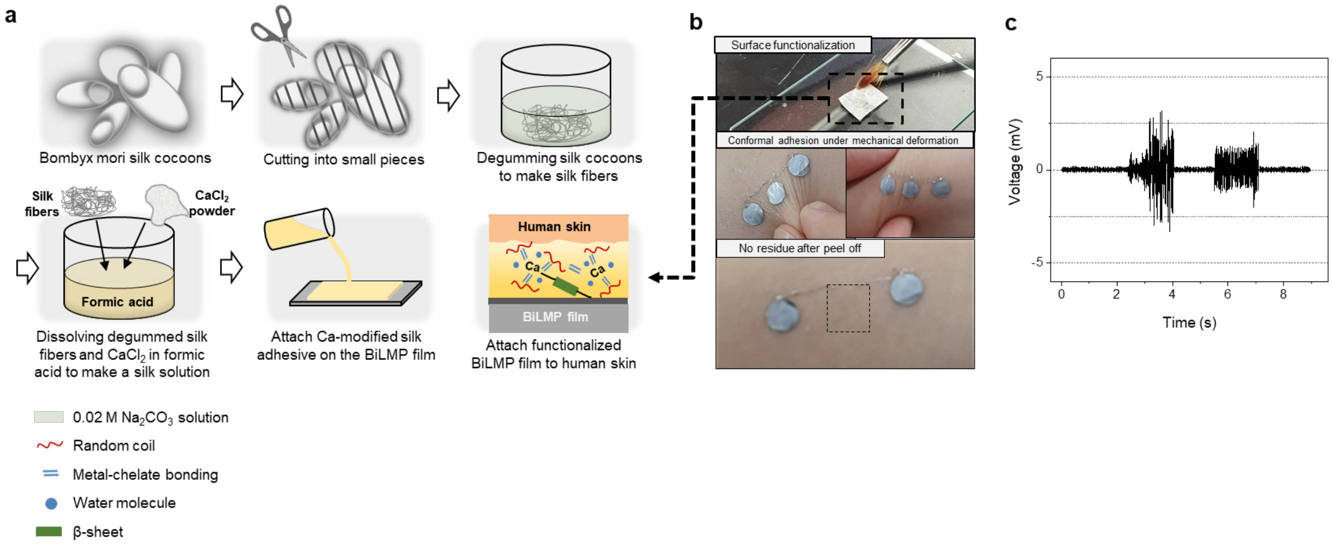

Supplementary Fig. 25| Functionalization of BiLMP film and its application as bioelectrode. **a**, Schematic illustration of functionalization of BiLMP film with silk adhesive. **b**, Photograph of functionalization of BiLMP with silk adhesive and its application as EMG sensor. **c**, Real-time EMG signal collected with functionalized BiLMP electrode.

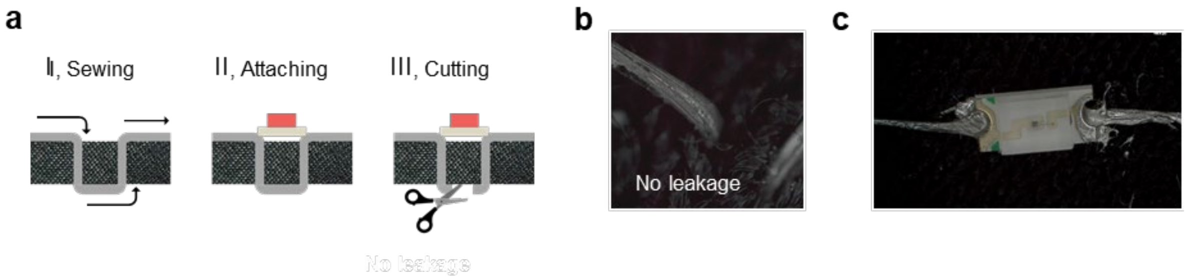

Supplementary Fig. 26| Integration of BiLMP fiber with clothing and electronics. **a**, Schematic illustration of sewing BiLMP fiber with clothes and integration of electronics. **b**, Optical microscope image of sewn BiLMP fiber. There are no leakage during sewing. **c**, Optical microscope image of LED integrated with BiLMP fiber.

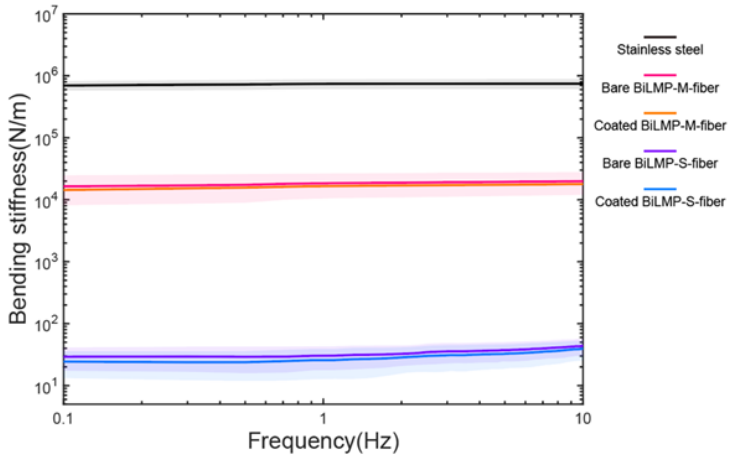

Supplementary Fig. 27| Stiffness of each fiber type.

Bending stiffness was measured with three samples (n=3).

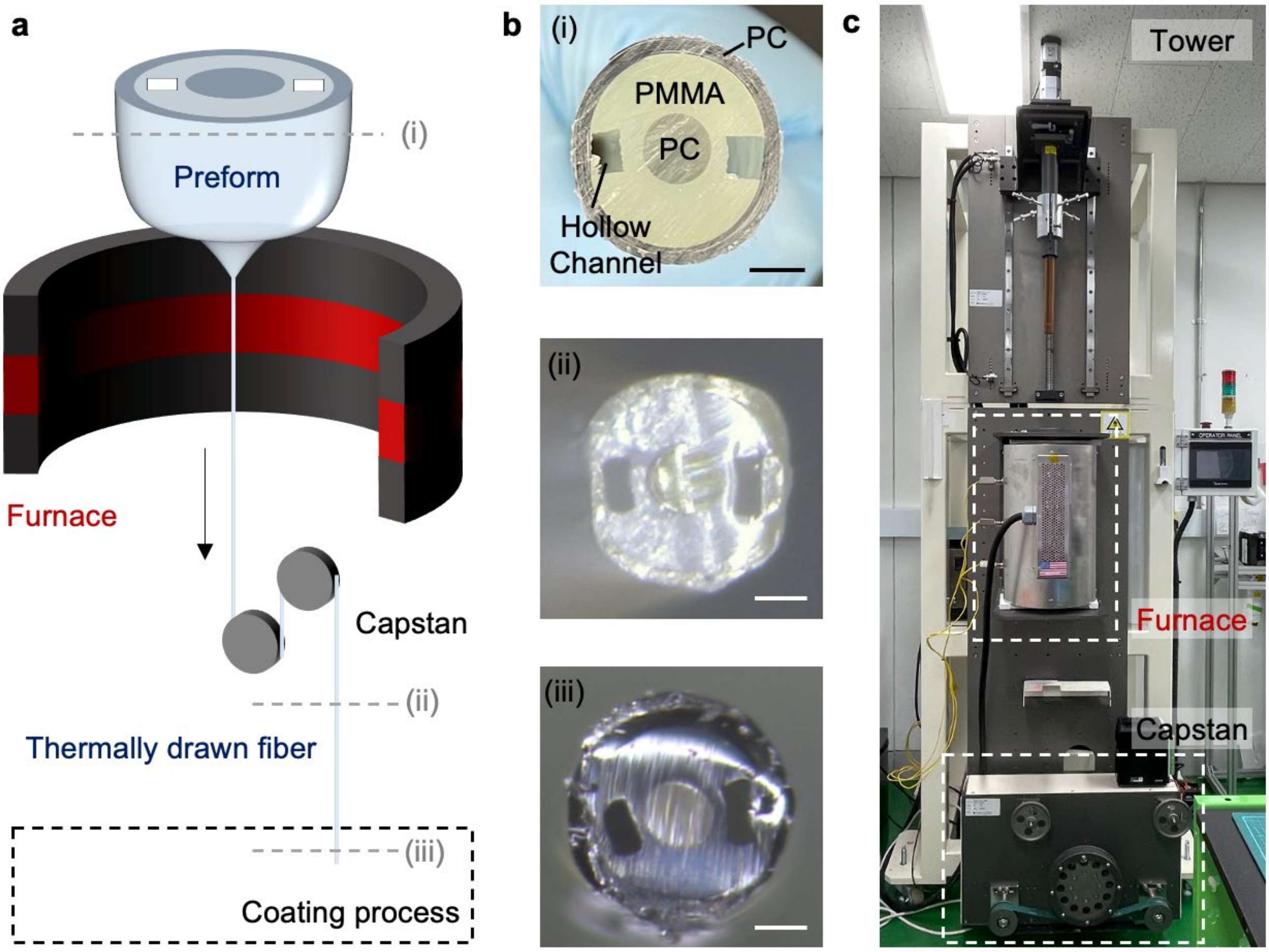

Supplementary Fig. 28| Thermally drawing process of multifunctional polymer fiber. a, Schematic illustration of TDP. **b**, Cross section image of (i) preform. (ii) thermally drawn fiber. (iii) BiLMP coated multifunctional fiber. Reproducibility: Suspension shearing was conducted six times, and on each occasion, it resulted in a compact coating. The scale bars represent 5mm, 50μm and 50μm. **c**, Photograph image of tower.

Thermally drawing process was used to fabricate micro scale multifunctional fiber. This fabrication process is advantage to mass production while scaling down with the same cross section (meter long and 200μm diameter fiber). It is possible to give multi functionality through the complex structure of preform. The hollow channel and PC/PMMA concentric structure of preform was utilized for microfluidic channel and waveguide, respectively. Thermally drawn fiber was encapsulated with a very thin elastomer, such as SEBS or PDMS, and coated with BiLMP as an electrode and PDMS as an insulator. This design allowed for specific electrophysiological recording at the fiber tip.

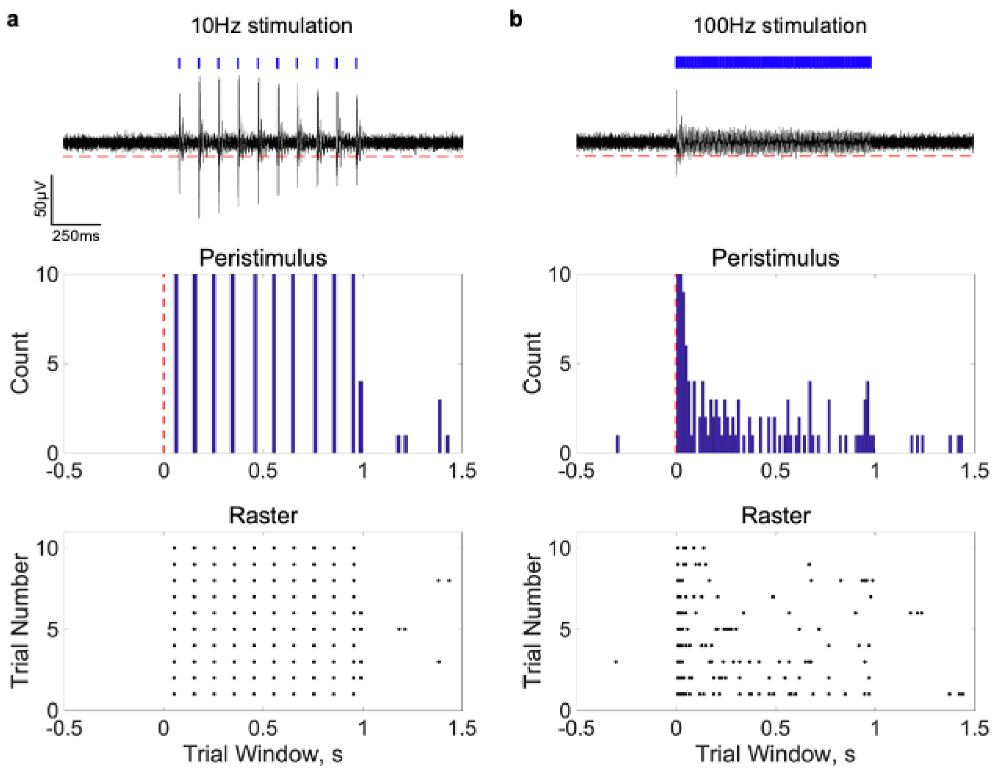

Supplementary Fig. 29| Recording of optically evoked potential with BiLMP based multifunctional fiber. **a**, 10Hz optical stimulation. **b**, 100Hz optical stimulation.

Optically evoked potential streams, peristimulus time histogram(PSTH), and raster plot of 10 trials. The evoked potential could not follow high frequency of optical stimulation even with sufficient stimulation power.

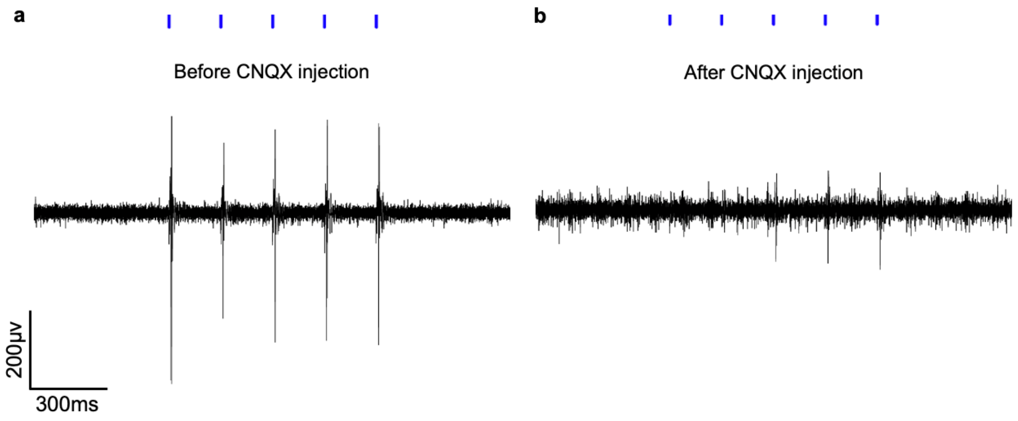

Supplementary Fig. 30| Neural activity in response to optical stimulation. a, Before drug injection **b**, After drug injection**.**

The 0.1mM CNQX in ACSF solution (5ul, 33nl/sec) is injected for demonstrating the functionality of drug delivery.

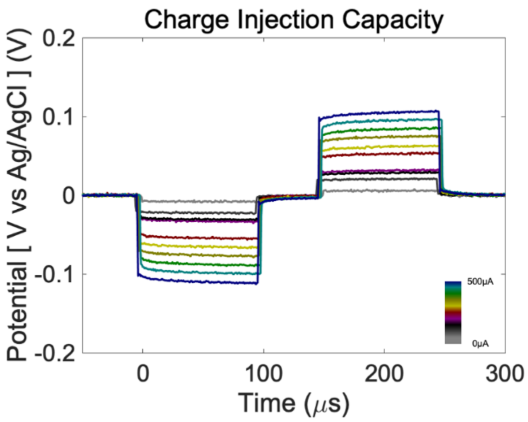

Supplementary Fig. 31| Charge injection capacity of BiLMP based stimulation thread.

The potential in response to biphasic current of 50 to 500μA (0.011A/cm^2 to 0.11 A/cm^2) indicates neuromodulation capability and safety of stimulation thread. The device has 400 Ohms impedance at 1kHz.

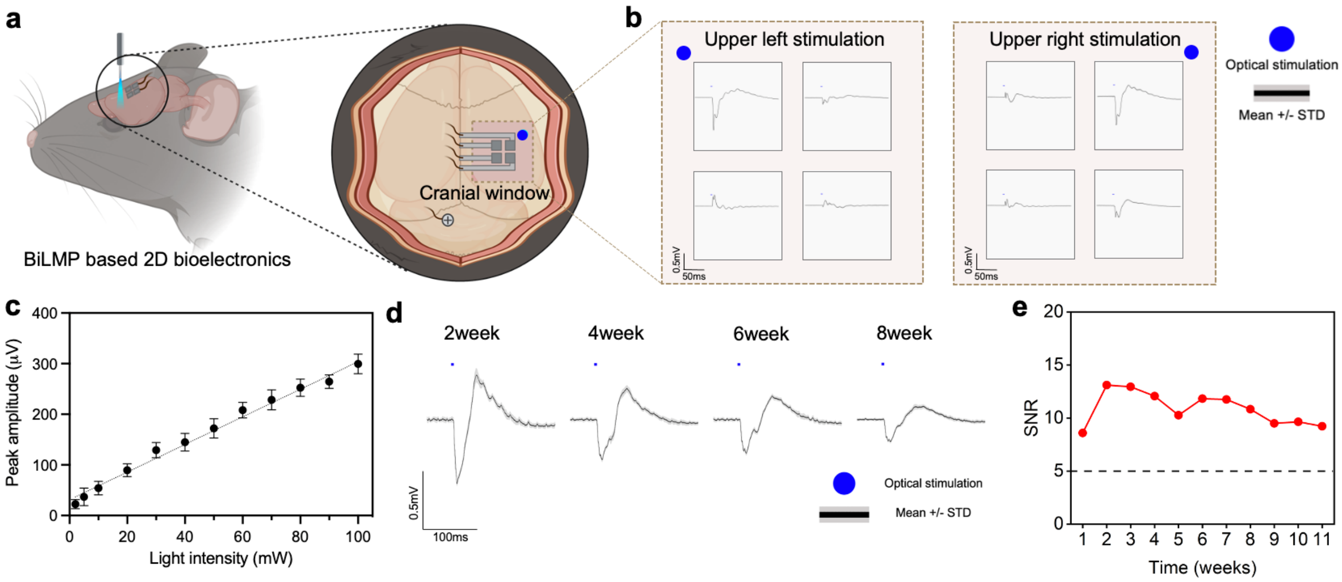

Supplementary Fig. 32| Chronic usability of BiLMP based bioelectronics. **a**, Schematic illustration of mounted BiLMP deposited ECoG device and neural recording procedure with photo stimulation. **b**, Recorded optically-evoked ECoG signal from mouse brain cortex. Optical stimulation is located upper left site or upper right site. Averaged 17 trials and 18 trials, respectively. **c**, Peak amplitude of optically evoked potential by light intensity. The values represent the mean and the standard deviation. Each 200 peaks are used in sampling. **d**, Prolonged recording capability from BiLMP-based devices. Averaged 100 trials. **e**, Signal to Noise Ratio of prolonged optically evoked potential. SNR lasted 11 weeks. Figure 32a was created with BioRender.com

To validate the chronic application of BiLMP-based electrodes, BiLMP-based array-type devices were fabricated. The device deposited on the brain cortex of *Thy1::ChR2-YFP* mice showed that they could successfully record the optically-evoked potentials in response to 473nm optical stimulation. As expected, the amplitude of signal recorded at the closest electrodes with stimulation site had a maximum value, and the peak amplitude of potentials have a linear correlation with light intensity, which is from the characteristics of optogenetic electrophysiology. In the long-term chronic experiments, it is confirmed that the recorded signal lasted until a few months after device implantation, which is from the high flexibility and biocompatibility of the BiLMP-based devices. Signal-to-noise ratio (SNR) of the recorded signal is maintained (~10) until 11 weeks after device implantation, while both peak amplitude and noise level gradually decreased.

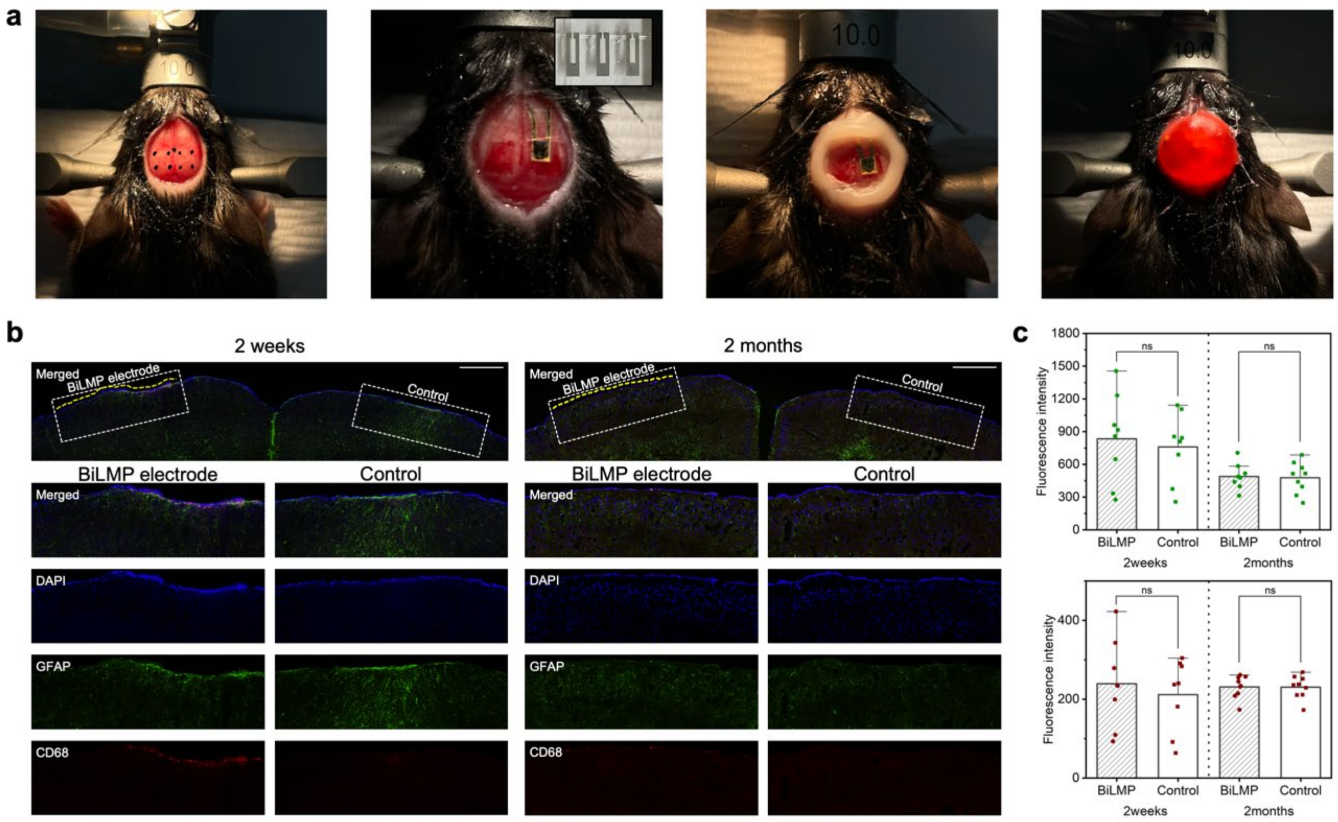

Supplementary Fig. 33| *In vivo* biocompatibility of BiLMP electrode. a, Surgical procedure for *in vivo* biocompatibility test. **b**, Representative confocal images indicating foreign body responses of mouse brain cortex with/without (left/right) BiLMP-based devices. Reproducibility: Immunohistochemistry staining was conducted in 17 brain slices, and each occasion is well stained. Scale bars represent 500μm. **c**, Expression of GFAP and CD68 after 2 weeks and 2 months implantation. Data are presented as mean values +/- SD. (n=8 samples at 2 weeks for each device and mouse. n=9 samples at 2 months for each device and mouse). The statistical analysis is two-sided test. GFAP, 2 weeks: P=0.6850, t=2.1447, 2 months: P=0.8495, t=2.1199, CD68, 2 weeks: P=0.5916, t=2.1447, 2 months: P=0.9538, t=2.1199, d.f. = 14 for all 2 weeks, d.f. = 16 for all 2 months. (N.S. P > 0.05, two sample t test).

To evaluate the biocompatibility of BiLMP-based electrodes, we characterized the expression of markers of foreign body response: glial fibrillary acidic protein (GFAP) for astrocyte and cluster of differentiation 68 (CD68) for activated macrophages near the surface of brain slice covered by the BiLMP based 2D substrate. For the comparison, cranial window at the contralateral lobe was used as a control group. The quantitative analysis for both markers 2 weeks and 2 months following the surgeries confirmed that there was no significant difference between groups for every timepoint, indicating that BiLMP electrodes hardly evoked the immune response.

References

1) Jiang, Z., Nayeem, M. O. G., Fukuda, K., Ding, S., Jin, H., Yokota, T., ... & Someya, T. (2019). Highly stretchable metallic nanowire networks reinforced by the underlying randomly distributed elastic polymer nanofibers via interfacial adhesion improvement. Advanced Materials, 31(37), 1903446.

2) Park, M., Im, J., Shin, M., Min, Y., Park, J., Cho, H., ... & Kim, K. (2012). Highly stretchable electric circuits from a composite material of silver nanoparticles and elastomeric fibres. *Nature nanotechnology*, *7*(12), 803-809.

3) Jin, H., Nayeem, M. O. G., Lee, S., Matsuhisa, N., Inoue, D., Yokota, T., ... & Someya, T. (2019). Highly durable nanofiber-reinforced elastic conductors for skin-tight electronic textiles. *ACS nano*, *13*(7), 7905-7912.

4) Xu, F., & Zhu, Y. (2012). Highly conductive and stretchable silver nanowire conductors. *Advanced materials*, *24*(37), 5117-5122.

5) Matsuhisa, N., Kaltenbrunner, M., Yokota, T., Jinno, H., Kuribara, K., Sekitani, T., & Someya, T. (2015). Printable elastic conductors with a high conductivity for electronic textile applications. *Nature communications*, *6*(1), 7461.

6) Yi, F. L., Meng, F. C., Li, Y. Q., Huang, P., Hu, N., Liao, K., & Fu, S. Y. (2020). Highly stretchable CNT Fiber/PAAm hydrogel composite simultaneously serving as strain sensor and supercapacitor. *Composites Part B: Engineering*, *198*, 108246.

7) Zhao, Y., Dong, D., Gong, S., Brassart, L., Wang, Y., An, T., & Cheng, W. (2019). A moss-inspired electroless gold-coating strategy toward stretchable fiber conductors by dry spinning. Advanced Electronic Materials, 5(1), 1800462.

8) Berteloot, G., Daerr, A., Lequeux, F., & Limat, L. (2013). Dip coating with colloids and evaporation. *Chemical Engineering and Processing: Process Intensification*, *68*, 69-73.

9) Landau, L., & Levich, B. (1988). Dragging of a liquid by a moving plate. In *Dynamics of curved fronts* (pp. 141-153). Academic Press.

10) Routh, A. F., & Zimmerman, W. B. (2004). Distribution of particles during solvent evaporation from films. *Chemical Engineering Science*, *59*(14), 2961-2968.

11) Cardinal, C. M., Jung, Y. D., Ahn, K. H., & Francis, L. F. (2010). Drying regime maps for particulate coatings. *AIChE journal*, *56*(11), 2769-2780.

12) Baesch, S., Price, K., Scharfer, P., Francis, L., & Schabel, W. (2018). Influence of the drying conditions on the particle distribution in particle filled polymer films: Experimental validation of predictive drying regime maps. *Chemical Engineering and Processing-Process Intensification*, *123*, 138-147.

13) Majumder, M., Rendall, C., Li, M., Behabtu, N., Eukel, J. A., Hauge, R. H., ... & Pasquali, M. (2010). Insights into the physics of spray coating of SWNT films. *Chemical Engineering Science*, *65*(6), 2000-2008.

14) Palma, S., & Lhuissier, H. (2019). Dip-coating with a particulate suspension. *Journal of Fluid Mechanics*, *869*, R3

15) Goldstein, R. J., Chiang, H. D., & See, D. L. (1990). High-Rayleigh-number convection in a horizontal enclosure. *Journal of Fluid Mechanics*, *213*, 111-126.

16) Wang, Q., Liu, H. R., Verzicco, R., Shishkina, O., & Lohse, D. (2021). Regime transitions in thermally driven high-Rayleigh number vertical convection. *Journal of fluid mechanics*, *917*, A6.

17) Lee, G. H. et al. A Personalized Electronic Tattoo for Healthcare Realized by On-the-Spot Assembly of an Intrinsically Conductive and Durable Liquid-Metal Composite. Advanced Materials 34, doi:10.1002/adma.202204159 (2022).

18) Park, Y.G et al. "Three-dimensional, high-resolution printing of carbon nanotube/liquid metal composites with mechanical and electrical reinforcement." Nano letters 19.8 (2019): 4866-4872.
